# A rare case report about a congenital adrenal hyperplasia by 21-hydroxylase lock in its pure virilizing form discovered in adolescence

**DOI:** 10.1016/j.amsu.2022.103673

**Published:** 2022-04-28

**Authors:** Achwak Alla, Najat Draoui, Imane Rami, Siham Rouf, Hanane Saadi, Imane Kamaoui, Hanane Latrech

**Affiliations:** a Department of Endocrinology-Diabetology, Mohammed VI University Hospital Centre, Faculty of Medicine and Pharmacy, Mohammed I University, Oujda, Morocco; b Laboratory of Epidemiology, Clinical Research and Public Health, Faculty of Medicine and Pharmacy, Mohammed I University, Oujda, Morocco; c Department of Obstetrics and Gynecology, Mohammed VI University Hospital Centre, Faculty of Medicine and Pharmacy, Mohammed I University, Oujda, Morocco; d Department of Radiology, Mohammed VI University Hospital Centre, Faculty of Medicine and Pharmacy, Mohammed I University, Oujda, Morocco

**Keywords:** Case report, Congenital adrenal hyperplasia, 21-Hydroxylase deficiency, Pure virilizing form, CAH, Congenital adrenal hyperplasia, CYP21, the gene encoding 21-hydroxylase, a cytochrome P-450 enzyme, SDHEA, Dehydroepiandrosterone Sulfate, CMIA, Chemiluminescence microparticle immunology, 17OHP, 17 hydroxyprogesterone, PCR, Polymerase Chain Reaction, ACTH, Adrenocorticotropic Hormone, CPA, Cyproterone Acetate

## Abstract

**Introduction:**

Congenital adrenal hyperplasia (CAH) due to 21-hydroxylase deficiency is an autosomal recessive disease. The diagnosis of the classic virilizing form must be made at birth.

**Case presentation:**

We report the case of a 16-year-old female patient, who consulted for primary amenorrhea and absence of breast development, in whom the clinical examination found a male morphotype, signs of virilization with a peniform hypertrophy of the clitoris. Pelvic ultrasound confirmed the presence of the uterus and ovaries. A syacthen test on 17 hydroxy-progesterone was performed confirming the diagnosis of congenital adrenal hyperplasia by partial 21-hydroxylase deficiency. The treatment was based on hydrocortisone and spironolactone with a decrease in hairiness and a breast development after 3 months.

**Discussion:**

The principal aim of the management at adolescent age is to block hyperandrogenism and to prevent or manage the complications of classic form and its treatment. The treatment must be completed by a feminization surgery which constitutes a great challenge given the necessity of participation of a gynecologist and a pediatric surgeon experienced in the surgery of anomalies of sexual development.

**Conclusion:**

This rare case of anomaly of sexual development discovered at an adolescent age with all the obstacles and difficulties of its management allows to put the point on the necessity of a good clinical examination at birth and the early management of any anomaly of sexual development.

## Highlights

• Discovery of congenital adrenal hyperplasia on 21-hydroxylase block in its pure virilizing form at a late age.
• Therapeutic management, especially surgery, is a great challenge at this late age.
• Treatment with hydrocortisone and spironolactone has led to clinical and biological improvement.

## 1 Introduction

Congenital adrenal hyperplasia (CAH) is one of the most common autosomal recessive disorders, it is potentially lethal in its classic form. The most common type of CAH is 21-hydroxylase deficiency. Females with classic 21-hydroxylase deficiency are exposed to excess androgens prenatally and are born with virilized external genitalia. The disease is caused by mutations in the CYP21 gene encoding the steroid 21-hydroxylase enzyme [1]. We report in this article, the rare case of an adolescent girl who has just been diagnosed with congenital adrenal hyperplasia in its pure classical virilizing form. This case has been reported following the CARE criteria [2].

## 2 Case presentation

**Fig. 1:**
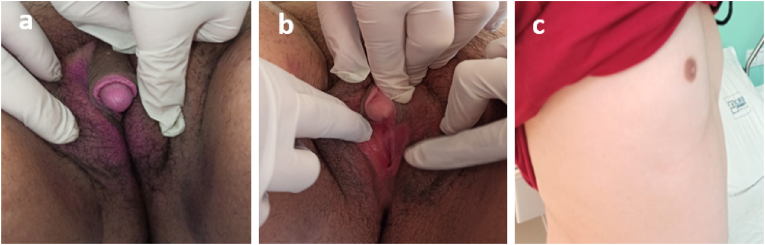
a-b) Genital examination before starting therapy showing a non-fused smooth pigmented and symmetrical genital folds, a clitoromegaly with peniform aspect and two separate orifices below the clitoris. c) Profile image showing absence of breast development Tanner stage 1.

The patient aged 16 years 8 months old, from a non-consanguineous marriage. The analysis of her history did not find maternal exposure to androgens during pregnancy or salt loss syndrome. Consulted us for the first time of her life for abnormal sexual development and primary amenorrhea whose history goes back to birth when her mother discovered the presence of a genital bud. The patient did not consult until puberty for lack of breast development and a primary amenorrhea with hirsutism. The clinical examination had objectified a male morphotype with lack of breast development S1 of Tanner. The genital examination revealed non-fused smooth pigmented and symmetrical genital folds, a clitoromegaly with peniform aspect measuring approximately 4.5 cm in length and 2 cm in width, two separate orifices below the clitoris (Prader II), with absence of gonad palpation at the level of the folds and at the inguinal level (Fig. 1). She had severe hirsutism with a subjective Ferriman and Galleway Score of 29 associated with signs of virilization.

The biological exploration showed a karyotype 46, XX on 50 mitoses, testosterone level 3.69 ng/ml (CMIA), SDHEA 752.5 μg/dl (CMIA), 17OH Progesterone after synacthene stimulation T60 min: 354 ng/ml (VN < 10 ng/ml, radioimmunology), the cortisol level was low at 62 g/ml (CMIA). Pelvic ultrasound revealed a hypoplastic uterus measuring 45 × 16 mm with regular contours, macropolycystic ovaries measuring 24 mm on the right, 22 mm on the left. Abdominal scan showed adrenal glands without abnormalities.

Therapeutically, hydrocortisone replacement at a dose of 20 mg/day was prescribed, as well as anti-androgenic treatment with spironolactone at a dose of 100 mg/day.

**Fig. 2:**
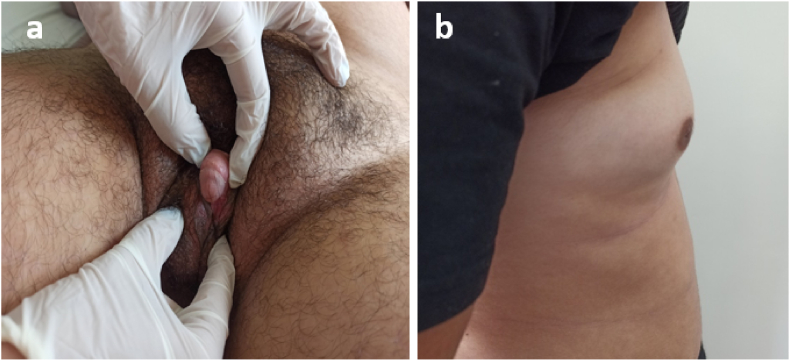
a) Genital examination 3 months after the beginning of therapy showing a decrease of the size of the clitoris in length measuring 3.5 cm vs 4.5 cm. b) Profile image showing the beginning of breast development Tanner stage 2.

3 months later, she had noticed a decrease in the frequency of hair removal. The clinical examination noted a slight decrease in hirsutism, Ferriman and Galleway score at 25 vs 29, the size of the clitoris has decreased in length measuring 3.5 cm vs 4.5 cm with the beginning of breast development stage S2 of Tanner (Fig. 2). On the biological level the testosteronemia has decreased to 1,12 ng/ml Vs 3,69 ng/ml, the 17 OHP returned to 168,6 ng/ml.

At 6 months of treatment, the patient became Tanner stage 4, biologically she had a testosteronemia of 3.06 ng/ml and a 17 OHP of 220 ng/ml, then we increased the dose of hydrocortisone to 25 mg/day.

At 9 months, the Ferriman and Galleway score was rated at 21, she was S5 at Tanner's stage. The testosteronemia decreased to 1.64 ng/ml and the 17 OHP to 163.9 ng/ml. We put her on progestin to stimulate menstruation. A clitoral plasty was proposed to the patient but she refused. The genetic molecular could not be performed due to the patient's financial problems.

## 3 Discussion

Congenital adrenal hyperplasia is a group of autosomal recessive disorders that occur as a result of complete or partial defects in one of several steroidogenic enzymes involved in the synthesis of cortisol from cholesterol in the adrenal glands. More than 95–99% of all cases of CAH are due to a deficiency of steroid 21-hydroxylase, an enzyme encoded by the CYP21A2 gene [3]. On the basis of data from millions of newborns screened worldwide, classic CAH occurs in 1 in 10,000 to 1 in 20,000 live births [4,5]. Non classic CAH is common worldwide, with an estimated prevalence ranging from 1 case per 200 persons to 1 case per 1000 persons [6].

Mutations in CYP21A2 (the gene encoding 21-hydroxylase, a cytochrome P-450 enzyme) result in lack of 21-hydroxylase, which is required for the production of cortisol and aldosterone in the adrenal cortex. A deficiency of this enzyme has cascading effects. Reduced cortisol leads to overproduction of pituitary corticotropin, which is responsible for the hyperplasia of the adrenal cortex and the increased secretion of cortisol precursors, in particular 17OHP and adrenal androgens, the main one being D4-androstenedione. This androgen can then be metabolized into testosterone and then into dihydrotestosterone in the target cells [7].

The genetic mechanisms responsible for 21-OH deficiency are directly related to a complex genomic organization. More than 200 mutations in the CYP21A2 gene have been reported, but about 10 mutations are responsible for more than 90% of HCS cases, either by gene conversion or unequal recombination. 70–75% of cases are due to point mutations in the CYP21A2 gene. 20% of cases are due to large deletions linked to a mechanism of unequal recombination or aberrant segregation during meiosis. In 1–2% of cases, 21-OH deficiency is associated with de novo mutations. Molecular diagnosis is performed by real-time quantitative PCR, using different pairs of primers specific for the CYP21A2 gene and not for the CYP21A1P pseudogene, followed by sequencing, to identify point mutations [8].

Classic form is represented by two phenotypes: simple virilizing (SV) and salt wasting (SW). The clinical presentation differs according to the age of discovery, sex and form of HCS. All patients with classic 21-OHD have ambiguous external genitalia [9]. In most cases, the diagnosis of the classic pure virilizing form is made at birth, in rare cases, such as our patient, the diagnosis is made late in childhood, adolescence or adulthood. Hyperandrogenism being a disturbing factor of the gonadotropic axis, will cause dysovulation or anovulation resulting in cycle disorders, amenorrhea or even infertility [10]. The diagnosis should be evoked in any patient presenting with hyperandrogenism and/or oligomenorrhea. In our case, the clinical presentation was very suggestive with severe hirsutism, male morphotype, and primary amenorrhea, absence of breast development and peniform appearance of the clitoris. The uterus, fallopian tubes and ovaries form normally. Pelvic ultrasound confirms the presence of the female genitalia and frequently finds an ultrasound appearance of micropolycystic ovaries secondary to hyperandrogenism, which should not be confused with micropolycystic ovary syndrome, which is a diagnosis of elimination. In our case, pelvic ultrasound revealed macropolycystic ovaries: on the right 24 mm, on the left 22 mm.

The standard for confirming the diagnosis of CAH remains serum 17-hydroxyprogesterone, most often with stimulation by synthetic ACTH. Thus, a baseline 17 OH progesterone value higher than 2 ng/ml or a concentration >10 ng/ml in the synacthen test confirms the diagnosis [4]. CYP21A2 genotyping is considered a valuable adjunct to biochemical analyses in the diagnosis of the disease [3]. The genetic study could not be performed due to the patient's financial problems**.**

The management of the classic form in adolescence and adulthood has a double objective: to block hyperandrogenism and to prevent or manage the complications of classic form and its treatment [11]. Hydrocortisone is the most commonly used glucocorticoid. There are other glucocorticoids with longer half-lives, such as prednisone, prednisolone, or dexamethasone, which provide stability of replacement effect throughout the day. Unfortunately, it is difficult to achieve androgen secretion suppression without a high dose of glucorticoides and consequently the risk of iatrogenic hypercortisolism is high. Until now, regardless of the regimen used, the dilemma persists between the use of physiological hydrocortisone, well tolerated but with poor control of androgen secretion, and long-acting glucocorticoids, with a higher risk of side effects. Recently, a new slow-release glucocoticoides formulation (Plenadren) has become available and another (Chronocort) is currently under investigation. This is another modified-release hydrocortisone formulation under development, administered twice daily at bedtime and upon awakening, and has been shown to mimic normal circadian cortisol levels, however, the drug has not yet been approved. A phase II trial demonstrated superior suppression of morning 17-OH progesterone levels (and, by inference, nighttime androgen secretion) in patients with congenital adrenal hyperplasia. Results of a Phase III trial are pending [12]. In our case, the treatment was based on the substitution of 20 mg/d of hydrocortisone in 2 doses.

To combat signs of hyperandrogenism, Cyproterone acetate (CPA) is a potent progestin that induces a decrease in plasma testosterone and delta4-androstenedione concentrations by inhibiting LH. It also blocks the peripheral effects of androgens by inhibiting their binding to their receptor. It is widely used in France and Europe within the framework of a marketing authorization for the treatment of hirsutism in women [11]. Only two randomized controlled studies with small numbers of patients have evaluated the efficacy of CPA, combined with an estrogen, and a glucocorticoid. In one of these, which included 30 patients, CPA combined with 17b estradiol showed superior efficacy on the Ferriman score compared with hydrocortisone (improvement in 54 versus 24% of cases). In patients in group 1, who received treatment with 20 mg hydrocortisone, hirsutism regressed slowly, In contrast, there was a striking drop in clinical score in group 2 treated with CPA with a difference in Ferriman score between the two groups that was already significant after 3 months of treatment (P < 0.05) and even more so after 6 and 12 months of treatment (P < 0.005) [13].

Spironolactone is an aldosterone antagonist that exerts anti- androgenic effects by blocking androgen receptors and also inhibits 5-α-reductase activity, its antiandrogenic effect is observed at doses ranging from 100 to 200 mg/day [11]. In our case, we opted for treatment with spironolactone 100 mg/day. After 3 months of well-conducted replacement therapy and anti-androgenic treatment, an improvement of the symptomatology was noted with a decrease in hirsutism, a decrease in the size of the clitoris and the beginning of breast development.

Fertility in women with a classical form of HCS due to 21-hydroxylase deficiency is diminished. This is the consequence of several factors, which are biological (poor hormonal balance), mechanical (related to reconstruction surgeries), psychological and sexual. Cycle disorders are thus frequent in the classical forms, from spaniomenorrhea to amenorrhea evaluated in 30–75% of those suffering from a pure virilizing form [14].

On the other hand, in severely virilized women, early surgery to repair the urogenital sinus should be discussed. In patients with congenital adrenal hyperplasia for whom surgery is chosen, vaginoplasty using urogenital mobilization as well as neurovascular-sparing clitoroplasty for severe clitoromegaly is considered [4]. In our case, surgery will be proposed and discussed with the patient after 6 months of medical treatment.

Mental health management is a valuable adjunct to endocrinological and surgical management. Case reports, but not systematic studies, have documented psychosocial consequences of atypical genital development. These consequences include awareness of the incongruity between the patient's genital appearance and assigned sex; conflicting gender identification by family members; and overall alteration of body self-image associated with short stature, increased weight, and hirsutism. These experiences can lead to social withdrawal, especially in situations involving nudity (team sports or medical examinations), and avoidance of romantic interactions and sexual engagement. As such, a mental and behavioral health consultation and assessment is recommended for patients and their families to address any concerns related to their illness. In addition, mental health practitioners should have specialized expertise in the assessment and management of psychosocial issues related to CAH [4].

Given the increased risk of obesity in adulthood, hyperandrogenism, poor statural growth and hyperinsulinemia, screening for these complications and management by hygienic-dietary measures must be implemented in these patients [15]. Thus, we noted an improvement in lipid and uric acid levels in our patient after 3 months of hygienic-dietary measures.

The limits of this case is the management, since the patient refused surgery, the medical treatment alone does not give a great effect on the clitoromegaly.

## 4 Conclusion

We report the case of a femal adolescent patient who consulted us for the first time in her life for anomaly of sexual development and primary amenorrhea in whom the diagnosis of HCS in its classic pure virilizing form was retained at this age, having required a specialized and complicated management. Hence the importance of making the diagnosis as early as possible to allow normal growth, female puberty and satisfactory fertility.

## Ethical approval

This is a case report that does not require a formal ethical committee approval. Data were anonymously registered in our database. Access to data was approved by the head of the department.

## Sources of funding

This research was not funded.

## Author contributions

Dr. Achwak Alla wrote the manuscript.

Dr. Najat Draoui helped in writing and literature review.

Dr. Imane Rami helped in writing and literature review.

Pr. Siham Rouf helped in writing, supervised the redaction and revised the manuscript.

Pr. Hanane Saadi helped in writin, gynecological exploration and revision of the manuscript.

Pr. Imane Kamaoui helped in writing, radiological exploration and revision of the manuscript.

Pr. Hanane Latrech helped in writing, supervised the redaction, revised and approved the final draft for publication.

All authors approved the final version of the manuscript.

## Registration of research studies

This is not an interventional study. We only reported the patient's findings from our database as a case report.

## Guarantor

Professor Hanane Latrech.

## Consent

A written informed consent was obtained from the parents of the patient for publication of this case report and accompanying images. A copy of the written consent is available for review by the Editor-in-Chief of this journal on request.

## Provenance and peer review

Not commissioned, externally peer reviewed.

## Declaration of competing interest

The authors declare no conflicts of interest.

## References

[1] Speiser P.W., White P.C. (2003). Medical progres, congenital adrenal hyperplasia. N. Engl. J. Med..

[2] Gagnier J.J., Kienle G., Altman D.G., Moher D., Sox H., Riley D. (2013).

[3] Concolino P., Costella A. (2018). Congenital adrenal hyperplasia (CAH) due to 21-hydroxylase deficiency: a comprehensive focus on 233 pathogenic variants of CYP21A2 gene. Mol Diagnosis Ther [Internet].

[4] Speiser P.W., Arlt W., Auchus R.J., Baskin L.S., Conway G.S., Merke D.P. (2018). Congenital adrenal hyperplasia due to steroid 21-hydroxylase deficiency: an endocrine society* clinical practice guideline. Vol. 103. J. Clin. Endocrinol. Metab..

[5] Therrell B.L., Berenbaum S.A., Manter-kapanke V., Simmank J., Korman K., Prentice L. (2017).

[6] Hannah-Shmouni F., Morissette R., Sinaii N., Elman M., Prezant T.R., Chen W. (2017). Revisiting the prevalence of nonclassic congenital adrenal hyperplasia in US Ashkenazi Jews and Caucasians. Genet. Med..

[7] El-Maouche D., Arlt W., Merke D.P. (2017). Congenital adrenal hyperplasia. Lancet.

[8] Dumeige L., Bouvattier C., Lombès M. (2017).

[9] Riepe F.G., Sippell W.G. (2007). Recent advances in diagnosis, treatment, and outcome of congenital adrenal hyperplasia due to 21-hydroxylase deficiency. Rev. Endocr. Metab. Disord..

[10] Moran C., Azziz R., Weintrob N., Witchel S.F., Rohmer V., Dewailly D. (2006). Reproductive outcome of women with 21-hydroxylase-deficient nonclassic adrenal hyperplasia. J. Clin. Endocrinol. Metab..

[11] Bachelot A., Touraine P. (2014). Prise en charge à l’âge adulte des hyperplasies congénitales des surrénales par déficit en 21-hydroxylase. Presse Med..

[12] Stewart P.M. (2019). Modified-release hydrocortisone: is it time to change clinical practice?. J Endocr Soc.

[13] Birman P., Mowszowicz I., Clair F., Kuttenn F., Mauvais-jarvis P. (2015). in Late-Onset Adrenal Hyperplasia.

[14] Robin G., Decanter C., Baffet H., Catteau-Jonard S., Dewailly D. (2014). Déficits en 21-hydroxylase et infertilité féminine: de la physiopathologie à la prise en charge thérapeutique. Gynecol. Obstet. Fertil..

[15] Mooij C.F., Van Herwaarden A.E., Sweep F.C.G.J., Roeleveld N., De Korte C.L., Kapusta L. (2017). Cardiovascular and metabolic risk in pediatric patients with congenital adrenal hyperplasia due to 21 hydroxylase deficiency. J. Pediatr. Endocrinol. Metab..

